# Supplementary material for: Coagulation measures after cardiac arrest (CMACA)
Source: PLoS One. 2023 Jan 6;18(1):e0279653. doi: 10.1371/journal.pone.0279653 (PMC9821485; doi:10.1371/journal.pone.0279653)
Supplement: Attachment — Submitted filename: Response to Reviewers.docx [file pone.0279653.s002.docx]

Reply to Reviewer

**We suggest the Authors to add a paragraph summarizing the main findings focusing on the clinical view.** 
🡪 Thank you for your comments. I added the following sentences in the Discussion Section.

Clinically, an increase in D-dimer levels at admission reflects a hypercoagulable state during CA, and a decrease over time reflects systemic fibrinolysis. An increase in the D-dimer level at admission was correlated with time from collapse to ROSC, and the D-dimer level at 24 h was associated with poor neurological outcomes after OHCA, which means that the results of systemic fibrinolysis may be related to the neurological outcome. Although we cannot recommend the use of anticoagulation agents, further studies are needed to suggest an optimal treatment strategy for coagulation abnormalities after CA.

Reviewers' comments:

**Comments to the Author**

1. Is the manuscript technically sound, and do the data support the conclusions?

The manuscript must describe a technically sound piece of scientific research with data that supports the conclusions. Experiments must have been conducted rigorously, with appropriate controls, replication, and sample sizes. The conclusions must be drawn appropriately based on the data presented.

Reviewer #1: Yes

 2. Has the statistical analysis been performed appropriately and rigorously?

Reviewer #1: Yes

 3. Have the authors made all data underlying the findings in their manuscript fully available?

The [PLOS Data policy](http://www.plosone.org/static/policies.action#sharing) requires authors to make all data underlying the findings described in their manuscript fully available without restriction, with rare exception (please refer to the Data Availability Statement in the manuscript PDF file). The data should be provided as part of the manuscript or its supporting information, or deposited to a public repository. For example, in addition to summary statistics, the data points behind means, medians and variance measures should be available. If there are restrictions on publicly sharing data—e.g. participant privacy or use of data from a third party—those must be specified.

Reviewer #1: Yes

 4. Is the manuscript presented in an intelligible fashion and written in standard English?

PLOS ONE does not copyedit accepted manuscripts, so the language in submitted articles must be clear, correct, and unambiguous. Any typographical or grammatical errors should be corrected at revision, so please note any specific errors here.

Reviewer #1: No

 5. Review Comments to the Author

Please use the space provided to explain your answers to the questions above. You may also include additional comments for the author, including concerns about dual publication, research ethics, or publication ethics. (Please upload your review as an attachment if it exceeds 20,000 characters)

**Reviewer #1: Congratulations to the author for this study.
1. New markers like CT-IGFBP-4 has been identified as predictive novel biomarker of Ischemic Cardiovascular events.**

**https://www.ncbi.nlm.nih.gov/pmc/articles/PMC9420648/. Please look into this study and discuss this vital biomarker in your discussion.**

🡪 Thank you for your comments. I added the following sentences in the Discussion Section.

When an ischemic cardiovascular event occurs, breakdown of insulin-like growth factor binding protein occurs, increasing the production of CT-IGFBP-4. CT-IGFBP-4 is known to be associated with the early diagnosis and outcomes of ischemic cardiovascular events. In this study, D-dimer at admission was not associated with outcomes after CA. Therefore, it is necessary to study whether CT-IGFBP-4, an early marker, is also useful in PCAS patients treated with TTM. [17]

17. Bhattarai A, Singh Sunar P, Shah S, Chamlagain R, Babu Pokhrel N, Khanal P, et al. CT-IGFBP-4 as a Predictive Novel Biomarker of Ischemic Cardiovascular Events and Mortality: A Systematic Review. Journal of Interventional Cardiology. 2022;2022:1-8. doi: 10.1155/2022/1816504.

**2. Lately, endothelium, platelets, and coagulation factors as the three vital components has been identified for diagnosing bleeding disorders (https://www.ncbi.nlm.nih.gov/pmc/articles/PMC9440837/). Please discuss the probable role of the coagulation that measured in cardiac arrest.**

🡪 Thank you for your comments. I added the following sentences in the Discussion Section.

Nevertheless, coagulofibrinolytic changes in PCAS patients treated with TTM are not fully understood. Endothelium, platelets, and coagulation factors are known as important components in the diagnosis of bleeding disorders and thus should be further studied in PCAS patients treated with TTM. [16]

16. Bhattarai A, Shah S, Bagherieh S, Mirmosayyeb O, Thapa S, Paudel S, et al. Endothelium, Platelets, and Coagulation Factors as the Three Vital Components for Diagnosing Bleeding Disorders: A Simplified Perspective with Clinical Relevance. International Journal of Clinical Practice. 2022;2022:1-10. doi: 10.1155/2022/5369001.

**3. The manuscript needs major English revision by a native speaker.**

🡪 Thank you for your comments. I received English proofreading by a native speaker at American Journal Experts. A proof of English proofreading is attached.

**4. Authors also needs to give recommendation related to findings of this study.**

🡪 Thank you for your comments. I added the following sentences in the Discussion Section.

An increase in D-dimer levels at admission reflects a hypercoagulable state during CA, and a decrease in D-dimer levels at 24 hours reflects systemic fibrinolysis. The hypercoagulable state during CA leads to the no-reflow phenomenon, leading to hypoperfusion of vital organs, including the brain. A large-scale randomized study with tissue plasminogen activator during CA showed no evidence of a beneficial effect. [31] However, there have been no studies on the effects of anticoagulant agents in PCAS patients with ROSC.

Clinically, an increase in D-dimer levels at admission reflects a hypercoagulable state during CA, and a decrease over time reflects systemic fibrinolysis. An increase in the D-dimer level at admission was correlated with time from collapse to ROSC, and the D-dimer level at 24 h was associated with poor neurological outcomes after OHCA, which means that the results of systemic fibrinolysis may be related to the neurological outcome. Although we cannot recommend the use of anticoagulation agents, further studies are needed to suggest an optimal treatment strategy for coagulation abnormalities after CA.

31. Abu-Laban RB, Christenson JM, Innes GD, Van Beek CA, Wanger KP, McKnight RD, et al. Tissue Plasminogen Activator in Cardiac Arrest with Pulseless Electrical Activity. New Engl J Med. 2002;346(20):1522-8. doi: 10.1056/nejmoa012885.

6. PLOS authors have the option to publish the peer review history of their article ([what does this mean?](https://journals.plos.org/plosone/s/editorial-and-peer-review-process#loc-peer-review-history)). If published, this will include your full peer review and any attached files.


If you choose “no”, your identity will remain anonymous but your review may still be made public.


**Do you want your identity to be public for this peer review?** For information about this choice, including consent withdrawal, please see our [Privacy Policy](https://www.plos.org/privacy-policy).

Reviewer #1: No
